# 

**Published:** 1881-03

**Authors:** 


### CONTENTS.

### COMMUNICATIONS.

Tooth Structure........................................ 89
Concerning the Newer Development of the Pathology of Nerves, and its Importance in Medical Instruction........................ 93
Synopsis of the Report of Analyses of Ohio River Water........................113
Pathology of the Osseous Structure of the Teeth........................116
Chemical Theory or Chemical Philosophy........................120
A German View of the Qualifications of a Physician........................126
Hot Water Compresses in Tetanus........................127
Trichinæ in a Cat........................127
Third Annual Meeting of the Alumni Association of the Ohio College of Dental Surgery........................128
Hospital Clinics........................129

### EDITORIAL.

Michigan Dental Society........................131
Ohio State Journal of Dental Science........................131
Certified........................132
A New Journal........................132

